# Three Years with the Army of the Potomac—A Personal Military History

**Published:** 1912-06

**Authors:** William Warren Potter

**Affiliations:** Buffalo, N. Y., Brevet Lieutenant Colonel, U. S. Volunteers; Surgeon in Charge of First Division Field Hospital Second Army Corps; Surgeon 57th Regiment New York Volunteers; Assistant Surgeon 49th Regiment New York Volunteers; Recorder Second Division Hospital Sixth Army Corps, etc., etc.


### Three Years with the Army of the Potomac—A Personal Military History

By William Warren Potter, M.D., Buffalo, N. Y., Brevet Lieutenant Colonel, U. S. Volunteers; Surgeon in Charge of First Division Field Hospital Second Army Corps; Surgeon 57th Regiment New York Volunteers; Assistant Surgeon 49th Regiment New York Volunteers; Recorder Second Division Hospital Sixth Army Corps, etc., etc.

1864.

Friday, January 1. A very cold and wintry day, with a raw, searching wind. I worked all day at the hospital, having chosen a site on the plain towards the Fitzhugh Homestead; but when General Hancock, who is on a visit to the Corps, got sight of the tents going up he interfered, saying it was not a safe place for the hospital; so I came back in the midst of a snowstorm, and pitched my tent for the night near Corps Headquarters. Thus, the hard work of New Year's Day went for naught. The next day was spent in choosing a site for the three hospitals of the corps, and I was out with Dr. Dougherty upon that business until near night. Finally, a spot was fixed upon in the woods along the Corduroy Road, about half way between Corps Headquarters and Brandy Station.

Monday, January 4. I took a large fatigue detail into the woods and commenced felling the trees, clearing the ground, and otherwise preparing it for the tents. The work is a laborious one, and will occupy us for some days before we shall be ready for the sick.

January 5. Still very busy at work getting the hospital in order. We have two sawmills within the lines of the first Division, on the banks of a small stream that furnishes the power to run them. They have been put in order by Captain Hoyt, Chief Quartermaster of the Division, and he has furnished me with a supply of lumber for hospital use. The tents are being floored with this lumber, and it comes in very conveniently for many other purposes also. This work went on for a week, when everything was declared in readiness for the reception of the sick. I had written my wife to start for Washington on the 12th, and that I would meet her there, having obtained permission for her to visit me for twenty days.

On the 13th. I went to Washington on a two days' leave, to meet her according to promise. She arrived late at night, and I found her in the dining room of the Metropolitan Hotel taking supper at eleven o'clock P. M., with Major Ellis, 49th New York, in whose company she had traveled from Buffalo. We remained in Washington during the 14th, and in the evening attended the theater (Ford's) where we met General Caldwell and wife. On the 15th, Thursday, we went down to the Army, and fairly established ourselves for the winter. I obtained a lady's saddle in Washington, and we were in the saddle together almost every day for the next two months. Mrs. Payne, Mrs. Arnold, Mrs. John Hancock, Mrs. Caldwell, Miss Jennie Kerner, and a few other ladies were our most intimate acquaintances in the First Division; but several ladies from the Second Division were our frequent guests.

The Third Corps issued invitations for a grand ball, to be given at General Carr's headquarters on January 25, and we received cards thereto on the 18th. We attended the ball on Monday, January 25, going over in an ambulance with Dr. and Mrs. Gesner. It was a successful affair in every way, and was attended by the ladies who were then present in the Army, and their husbands, as well as many other officers. The Hon. John Minor Botts, who lived in sight of the ball, attended with his two daughters. The supper, an elegant one, was sent down by special train from Washington.

### Morton's Ford.

On Saturday, February 6th, the Second Corps was ordered down to the Rapidan at Morton's Ford, and had a skirmish there with the enemy. The Third Division, commanded by General Alexander Hays, was thrown across the river, and had a severe skirmish with the force defending the Ford. This Division lost about two hundred men in killed and wounded. No orders came for me to go with the Division. Nor was the movement known at the hospital until the firing was distinctly heard. We then inquired and learned the cause. The wounded, chiefly of the Third Division, were brought up and distributed to the hospitals of the corps, and cared for by us until their recovery. This was the first instance, so far known, during the war where the wounded after a fight were treated from first to last in field hospitals. I made several operations of a capital order, and sent the specimens to Washington where they were deposited in the Army Medical Museum. One man died with lockjaw several days after the fight. He was wounded in the thigh, and the ball was impacted just above the outer condule of the left femur. At the autopsy it was discovered that the femur was fissured longitudinally, extending from the point of the impaction of the ball to the trocanters—an unusual wound. Mrs. Potter had an opportunity to assist in the care of these wounded soldiers, which she did with a kindly heart and thoroughly useful manner.

THE SECOND CORPS' BALL.

On Monday, February 22, Washington's Birthday, the Second Corps gave a grand ball at the headquarters of the corps near Stevensburg, or, to be more precise, at the Thom House on Cole's Hill. Adjoining the house a dancing hall was built, which required 12,000 feet of lumber to floor. This was seasoned pine borrowed of General Ingalls, Chief Quartermaster A. of P., which was smoothed and fitted by carpenters detailed from the ranks. The sides of the building were constructed of green hemlock which our sawmills furnished, and the roof was of canvas. It was lighted with 600 adamantine candles, the holders for which were especially made in Washington. The supper was furnished by Gautier, a Washington caterer, for which we paid him $2,200 and furnished transportation for it to the camp. It was a subscriptoin ball, no fees being taken for anything on that night, and no officer outside of the corps being allowed to subscribe; in other words, every visitor was considered the guest of the managers. Officers in the Second Corps who were solicited, subscribed according to rank or position, and my subscription was $20.00.

General Meade, General Sedgwick, General Pleasanton, and other high officers were present, besides Vice-President Hamlin and daughter, Governor Sprague and wife (nee Kate Chase) Senator Wilkinson, of Minn., and other distinguished civilians. The distinctive flags of the corps' and the camp, and garrison flags, were festooned in an artistic way about the room. An orchestra was built up across one end of the hall, upon which were mounted two brass Napoleon guns with the requisite ammunition, and three bands furnished uninterrupted music till morning. The pickets at the Rapidan were doubled that night, and a Brigade was sent down within supporting distance of the picket line, so as to be in readiness in case of a surprise; but, happily, there was no attack, and all went off in splendid style. This was, undoubtedly, the largest strictly miliary ball ever given in this country, a pleasure to the participants and a credit to the managers.

February 23. General Meade reviewed the Second Corps and Kilpatrick's Division of Cavalry, in honor of the visitors which the ball had attracted. It was a beautiful day, soft and balmy as spring, and the ladies attended in full force and fine array, many of them mounted upon fine horses, and many others in ambulances and spring wagons. Colonel Ulric Dahlgren, of the cavalry, a son of Admiral Dahlgren, was conspicuous that day as the only one-legged officer present, and he was noted for his excellent horsemanship, notwithstanding his physical imperfection. Alas! He was soon to meet his death, even within a few days, which occurred during Kilpatrick's celebrated raid to the environs of Richmond, which he started out upon the next day after the review.

At the first Division headquarters a fine music hall was constructed early in the season, and here we met almost every night for some sort of an entertainment, lectures, balls, and hops being the chief amusements. Grace Greenwood (Mrs. Lippincott) gave us three or four of her characteristic talks, that bristled with wit, wisdom, and genuine loyalty. The days were spent in riding, visiting, witnessing drills, and artillery practice; the evenings in cards, or at the entertainments at music hall. The ladies entered into all the ways and sports of camp life with a will, and before the season was over were really veterans in all the methods and habits of military life. During the winter about four thousand women visited the Army, under the generous permission of the commanding General, and I am sure it did both them and the Army much good.

I had my wife's visiting permit extended twice for twenty days each time, so that she was with me sixty days altogether. We had a fine quarters, a hospital tent 14 x 16 feet for the front room, and a regulation wall tent 9 x 9 feet for the sleeping apartment. These rooms were both warmed by stoves, carpeted with army blankets, and were very comfortable. I obtained a church door down at a cavalry picket on the Rapidan, and had it fitted as a front door to my tent, which was the envy of all our visitors, as well as the pride of ourselves.

One day, about the first of March, while General Caldwell and wife were dining with us, in walked our cousin, Milton G. Potter, then in his senior year at Rochester University, and now visiting the Army for recreation and information. He had been spending a few days with the cavalry on our right near Culpepper C. H., and was quite surprised to find everything so comfortable and homelike in our quarters. He joined us at dinner, and remained with us a few days, though it was only his purpose to make a short call when he came. I took him to Division Headquarters the next day, and there made him acquainted with all the officers, which his previous meeting with General Caldwell greatly facilitated and relieved of that stiffness incident to first introductions. We also visited the Sixth Corps and other points of interest during his visit, and he was so well pleased with the manner of the entertainment we provided that he prolonged his say to nearly a week, finally leaving with sincere expressions of regret. He wrote me afterwards how much he enjoyed his visit to the First Division, and this letter is preserved among my military papers, and so the winter wore away, all too rapidly to be sure, for those of us who were so pleasantly situated.

On March 12 I obtained a leave of absence for ten days to go home with my wife, and on a bright Monday morning, March 14, we bade adieu to the festive scenes of camp life, and turned our faces regretfully northward. From Washington we went to New York where we spent two days, reaching home on the 17th. After a week of rest, part of which was spent in visiting friends, I started back on the 24th, arriving in Elmira Friday morning, the 25th, just ten minutes too late for the train over the N. C. R. R. for Baltimore, which compelled me to wait twelve hours for the next one south, so I did not reach Washington until after mid-day Saturday.

Sunday, March 27. I started for the front this morning, reached the hospital this P. M., and found everything in good order, Dr. Rowland having been in charge during my absence. I found the Army in a sea of mud, from the effects of a ten inch snow-fall the previous Tuesday, which had now melted and left the surface of the ground in a liquid state. Dr. Aiken, in charge of the Second Division Hospital, is sick; otherwise everything is as usual about the whole camp. I learn that negatives of the hospitals have been taken by Gardner during my absence, and two pictures have been ordered for me, that are to be delivered very soon.

March 29. It has been raining hard all day, which will delay a movement of the Army for the present. General Grant has arrived at Culpepper C. H., where he has taken up his head-quarters. The Army has been re-organized, as has been contemplated for some time, and the Corps reduced to three, viz.: the Second, Fifth and Sixth, commanded by Hancock, Warren and Sedgwick, respectively. The Third Division of the Second Corps has been broken up and distributed to the First and Second Divisions; General Hays takes command of a Brigade in the Third Division (Birney's from the Third Corps); General Caldwell goes to Washington upon court martial duty; General Francis C. Barlow, formerly Colonel of the 61st New York, takes command of the First Division; Colonel Beaver's regiment, the 148th Pa., goes to the Fourth Brigade; General Webb goes back to a Brigade, and General Gibbon is assigned to the command of the Third Division. The Second Corps now has four divisions; two are made up of its own original three, and two come from the old Third Corps.

Dr. Dudley, 14th Conn., is now in the Second Division, and does not like the change at all; Dr. Gesner also goes to the

Second Division. Lieutenant Pelton is promoted to Captain, and is appointed Chief of Ambulances in Livermore's place. These are some of the changes that affect my friends or acquaintances. I visited the 57th yesterday, and found the officers all well excepting Dr. Nelley, who returned from his leave on Saturday, the 26th. Colonel Chapman has offered his resignation, but is doubtful about its acceptance, I imagine.

